# Effects of sex and estrous cycle on extended-access oxycodone self-administration and cue-induced drug seeking behavior

**DOI:** 10.3389/fnbeh.2024.1473164

**Published:** 2024-11-13

**Authors:** Bhumiben P. Patel, Jessica A. Loweth

**Affiliations:** Department of Cell Biology and Neuroscience, Schools of Osteopathic Medicine and Translational Biomedical Engineering & Sciences, Virtua Health College of Medicine & Life Sciences of Rowan University, Stratford, NJ, United States

**Keywords:** estrous cycle, estrous cycle dysregulation, oxycodone, incubation, drug seeking, sex differences

## Abstract

**Introduction:**

Increasing evidence indicates that sex is a factor that impacts the abuse liability and relapse vulnerability of prescription opioids like oxycodone. However, while women are more likely than men to be prescribed and to use these drugs, the impact of sex and ovarian hormones on prescription opioid use and relapse vulnerability remains unclear. Accurately assessing these measures is complicated by the fact that chronic opioid exposure can lower ovarian hormone levels and cause cycle irregularities.

**Methods:**

Adult male and female Sprague–Dawley rats self-administered oxycodone (0.1 mg/kg/infusion) under extended-access conditions (6 h/day, 10 days) followed by forced abstinence. Separate groups of animals received cue-induced seeking tests in a drug-free state during early (1–2 days) or later periods of abstinence (43–45 days). To track estrous cycle stage, animals were regularly vaginally swabbed throughout the study.

**Results:**

We observed oxycodone-induced estrous cycle dysregulation in the majority (~60%) of the animals during both self-administration and the first month of abstinence. In animals whose cycles were not dysregulated, we found a reduction in oxycodone intake during estrus compared to all other cycle stages (non-estrus). We also found that males but not females showed a time-dependent intensification or incubation of cue-induced oxycodone craving over the first 6 weeks of abstinence. This sex difference was estrous cycle-dependent, driven by a selective reduction in drug seeking during estrus.

**Discussion:**

These findings highlight the importance of tracking drug-induced estrous cyclicity and identify a clear impact of ovarian hormones on oxycodone taking and seeking behavior.

## 1 Introduction

Prescription opioid misuse has fueled the current opioid epidemic, which continues to grow at an alarming rate. Due to the reinforcing properties of prescription opioids like oxycodone, initial medical prescription opioid use can lead to misuse and dependence, including transitioning to more powerful opioids such as heroin or dangerous synthetic opioids such as fentanyl (Dickson-Gomez et al., 2022). High relapse rates further contribute to the opioid crisis, with some studies reporting that over half of recovering opioid-dependent users relapsed within the first week of abstinence (Woody et al., 2008; Smyth et al., 2010; Weiss et al., 2011). Sex is a biological factor known to influence prescription opioid use, misuse and craving. However, the impact sex has on these measures is complex. During the first “wave” of the opioid epidemic in the United States (1999 to 2010), prescription opioid-related overdose deaths increased at a dramatically higher rate in women (415%) compared to men (265%) (Mack et al., 2013). On the other hand, despite the fact that women were prescribed and used more prescription opioids than men (Goetz et al., 2021), analyses of data from 2017 found that men were more likely than women to report misuse of prescription opioids and to meet the criteria for drug dependence (Silver and Hur, 2020).

Preclinical rodent studies investigating sex differences in oxycodone taking and seeking behavior in rats have also yielded mixed results. Some report increased oxycodone intake in female compared to male rats under both intravenous (Mavrikaki et al., 2017; Kimbrough et al., 2020) and oral routes of oxycodone self-administration (Fulenwider et al., 2020). Others found no overall sex differences in oxycodone taking or seeking behavior (Bossert et al., 2020; Fulenwider et al., 2020; Hinds et al., 2023). However, these latter studies observed sex-specific effects of a mu opioid receptor agonist and a dopamine modulator on the intensification or incubation of cue-induced oxycodone seeking or craving that occurs during abstinence and is thought to reflect increased relapse vulnerability (Bossert et al., 2020; Fredriksson et al., 2020).

One factor known to influence sex differences in substance use disorders is fluctuations in levels of the ovarian hormones estradiol and progesterone that occur across the reproductive cycle in both humans and rodents (Carroll et al., 2004; Moran-Santa Maria et al., 2014; Carroll and Lynch, 2016; Knouse and Briand, 2021; Peart et al., 2022; Maher et al., 2023). However, assessing sex differences in prescription opioid abuse liability and relapse vulnerability is complicated by the fact that chronic opioid use can dysregulate the hypothalamic pituitary gonadal axis, leading to irregular hormonal fluctuations and cycles (Daniell, 2008; Rhodin et al., 2010; Vuong et al., 2010; Seyfried and Hester, 2012; Brennan, 2013). The extent of this dysregulation is likely to be influenced by both the dose and length of oxycodone exposure. For example, rats that self-administered oxycodone under short-access conditions (1 or 2 h) showed no signs of estrous cycle dysregulation (Mavrikaki et al., 2017; Vassoler et al., 2018; Hinds et al., 2023). However, studies conducted using longer-access intravenous oxycodone self-administration (≥ 6 h) either did not track the rodent reproductive (estrous) cycle (Bossert et al., 2020; Fredriksson et al., 2020; Kimbrough et al., 2020) or observed baseline estrous cycle dysregulation in their animals (Olaniran et al., 2023). It is therefore unclear how longer-access oxycodone exposure impacts gonadal hormone-dependent changes in estrous cyclicity in rodents and how these hormonal fluctuations, when intact, impact changes in oxycodone taking and seeking behavior. Here we address this gap by tracking and identifying oxycodone-induced estrous cycle dysregulation and by assessing oxycodone taking and time-dependent changes in cue-induced seeking behavior in males and females and during periods of regular estrous cyclicity. We focused on time-dependent changes in cue-induced oxycodone seeking or craving in which craving is low during the first few days of abstinence and progressively increases or incubates over time, reflective of increased relapse vulnerability (Grimm et al., 2001; Lu et al., 2004; Wolf, 2016). Due to oxycodone-induced estrous cycle dysregulation observed during the first month of abstinence, we assessed the impact of estrous cycle fluctuations on incubated craving following prolonged abstinence (days 43–45), when estrous cyclicity returned.

## 2 Materials and methods

### 2.1 Subjects and experimental design

**Figure 1:**
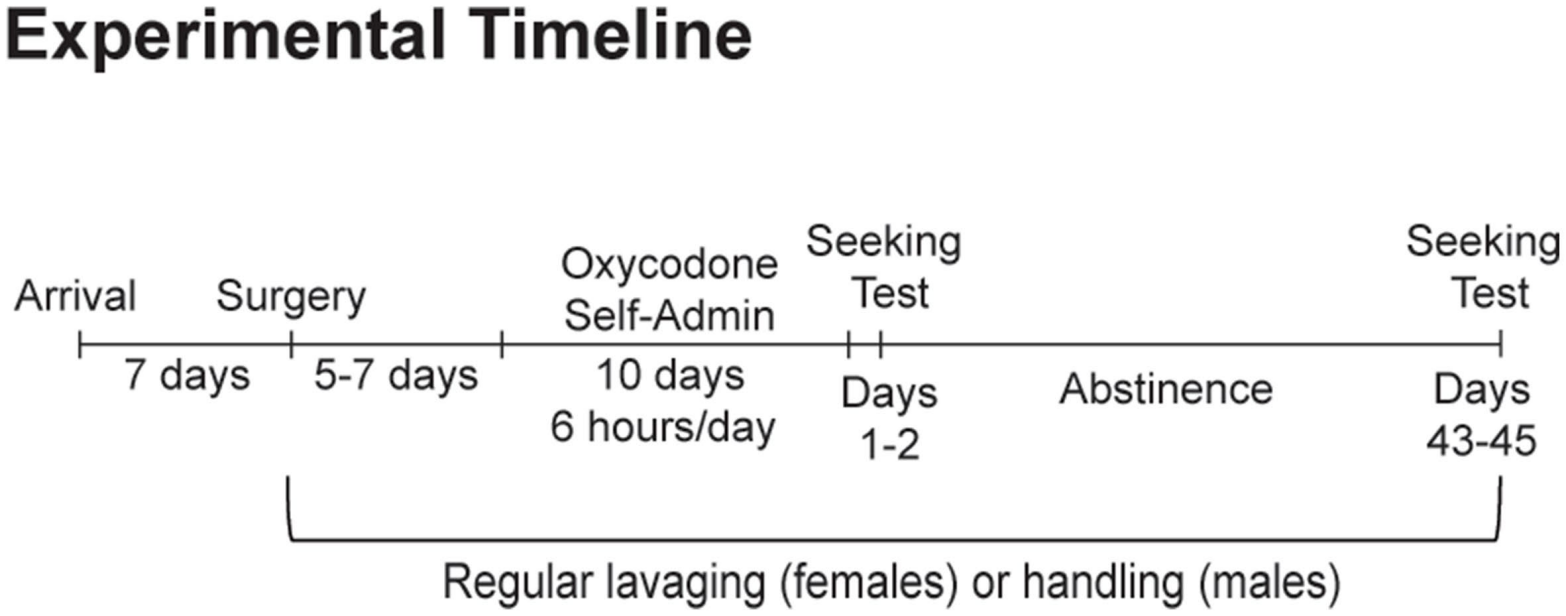
Experimental timeline. Separate groups of animals were tested for cue-induced oxycodone seeking on abstinence days 1–2 and days 43–45.

Adult male and female Sprague–Dawley rats were purchased from Envigo (Frederick, MD), with males weighing 250–275 grams and females weighing 225–250 grams upon arrival. Animals were housed on a reverse dark–light cycle (lights off at 9:00 AM, on at 9:00 PM) with *ad libitum* access to food and water. Rats were group housed by sex during an initial week-long acclimation period. Following this acclimation period and for the remainder of the study, regular vaginal swabbing began for female rats while males were handled on an identical schedule. Following survival surgery to implant an intravenous jugular catheter and sufficient recovery (5–7 days), animals self-administered oxycodone self-administration under extended-access conditions (0.1 mg/kg/infusion, 6 h/d for 10 d). Animals then underwent forced abstinence in their home cages. To assess the impact of sex and estrous cycle fluctuations on known time-dependent changes in cue-induced oxycodone seeking, one group of rats was tested during early abstinence, when craving is low (days 1–2; *n* = 8 males, *n* = 19 females). A separate group of rats was tested following prolonged abstinence (days 43–45; *n* = 11 males, *n* = 19 females), when craving has incubated. Due to estrous cycle dysregulation observed during the first month of withdrawal, the impact of estrous cycle fluctuations on oxycodone seeking was only assessed on days 43–45, when estrous cyclicity had returned in all animals (*n* = 19). Animals were excluded due to poor self-administration training (*n* = 3) and due to computer malfunctions during behavioral testing that prevented accurate data collection (*n* = 3). The number of rats reported refers to rats included in the statistical analyses. See Figure 1 for a graphical depiction of this experimental timeline.

### 2.2 Surgery

All animals underwent jugular catheter surgery as described previously (Glynn et al., 2018; Corbett et al., 2021; Munshi et al., 2021; Corbett et al., 2023a,b). Rats were anesthetized with ketamine & xylazine (Covetrus, Portland, ME; males: 80 mg/kg & 10 mg/kg, i.p.; females: 60 mg/kg & 7.5 mg/kg, i.p.) and a silastic catheter (Plastics One, Roanoke, VA) was first inserted into the right jugular vein and then passed subcutaneously to the mid-scapular region. Rats were singly housed immediately following surgery and for the remainder of the study. All animals were given 5 to 7 days to recover from surgery before oxycodone self-administration began. All procedures were approved by the Institutional Animal Care and Use Committee and conducted in accordance with the United States Public Health Service Guide for Care and Use of Laboratory Animals.

### 2.3 Estrous cycle monitoring

Estrous cycle was determined in intact, freely cycling females throughout the course of the experiment. Females were swabbed daily (~4–5 consecutive days with ~2–3 days off per week) at the onset of the dark cycle (~9:00 AM), approximately 30 min prior to the beginning of self-administration training or cue-induced seeking tests. Males were handled on an identical schedule. As described previously (Corbett et al., 2021; Corbett et al., 2023a,b), a cotton-tipped applicator dipped in saline (0.9%) was used to gently swab the vaginal canal and vaginal samples were placed on microscope slides and stained with the Papanicolaou (PAP) stain kit (Abcam, Waltham, MA). Vaginal samples were examined using light microscopy to determine each animal’s estrous cycle stage (metestrus or diestrus I, diestrus II, proestrus, estrus) and images were acquired using the Keyence BZ-X710 light/fluorescence microscope. Each stage of the cycle is defined by the absence, presence, or proportion of different cell types, with nucleated epithelial cells dominating during proestrus, anucleated cornified epithelial cells dominating during estrus, a mixture of nucleated epithelial cells, anucleated cornified epithelial cells, and leukocytes during metestrus or diestrus 1 and leukocytes dominating during diestrus II (Cora et al., 2015). In order for a stage to be assigned, the majority (>50%) of cells present on the slide must be representative of that stage (Kerstetter et al., 2008; Corbett et al., 2021). In normally cycling animals (those showing typical cycle length of 4–5 days and appropriate cycle phases), the stages immediately before and after each swab were also taken into account when determining or identifying each cycle stage.

### 2.4 Oxycodone self-administration

Following a sufficient recovery period (5–7 days), adult male and female Sprague–Dawley rats self-administered oxycodone (MilliporeSigma, Burlington, MA) under a fixed-ratio-1 (FR1) reinforcement schedule (0.1 mg/kg/infusion, 6 h/day for 10 days). Each session started shortly after the onset of the dark cycle (~9:30 AM) in operant chambers (MED Associates, St. Albans, VT) equipped with active and inactive nose-poke holes. As described previously, nose-pokes in the active hole responses turned on the infusion pump and led to the delivery of a 20 s light cue and a 20 s timeout period, while nose-pokes in the inactive hole were without consequence (Glynn et al., 2018; Corbett et al., 2021; Munshi et al., 2021; Corbett et al., 2023a,b). Animals that failed to stably self-administer oxycodone across the 10 days were excluded from the analyses (defined as obtaining less than an average of 20 infusions across the last 5 days of self-administration).

### 2.5 Cue-induced oxycodone seeking tests

Seeking tests were administered within 1 day of each time-point (days 1–2, days 43–45) in order to obtain enough animals across all of the four different stages (metestrus or diestrus I, diestrus II, proestrus, estrus) of the estrous cycle (4 to 5 days in length). Males were tested on an identical schedule (divided equally across days 1–2 or 43–45) in order to have an even distribution across these days within all groups. During the cue-induced seeking test, nose-pokes in the active hole resulted in presentation of the light cue previously paired with oxycodone but no drug infusion. Responding in the inactive hole had no consequence and serves as a control for general activity level. The number of times an animal responds in the active hole in this drug-free state provides the operational measure of cue-induced drug seeking or craving (Grimm et al., 2001).

### 2.6 Statistical analyses

Self-administration and time-course seeking test data were analyzed using between-within ANOVAs with sex or cycle as the between-subjects factor and self-administration day or time as the within-subjects factor. Seeking test data across both time-points were analyzed using two-way ANOVAs with sex or cycle and abstinence day as the between-subjects factors. In some cases, unpaired *t*-tests or one-way ANOVAs were used to compare self-administration or seeking behavior between specific groups.

## 3 Results

### 3.1 Oxycodone-induced estrous cycle dysregulation

**Figure 2:**
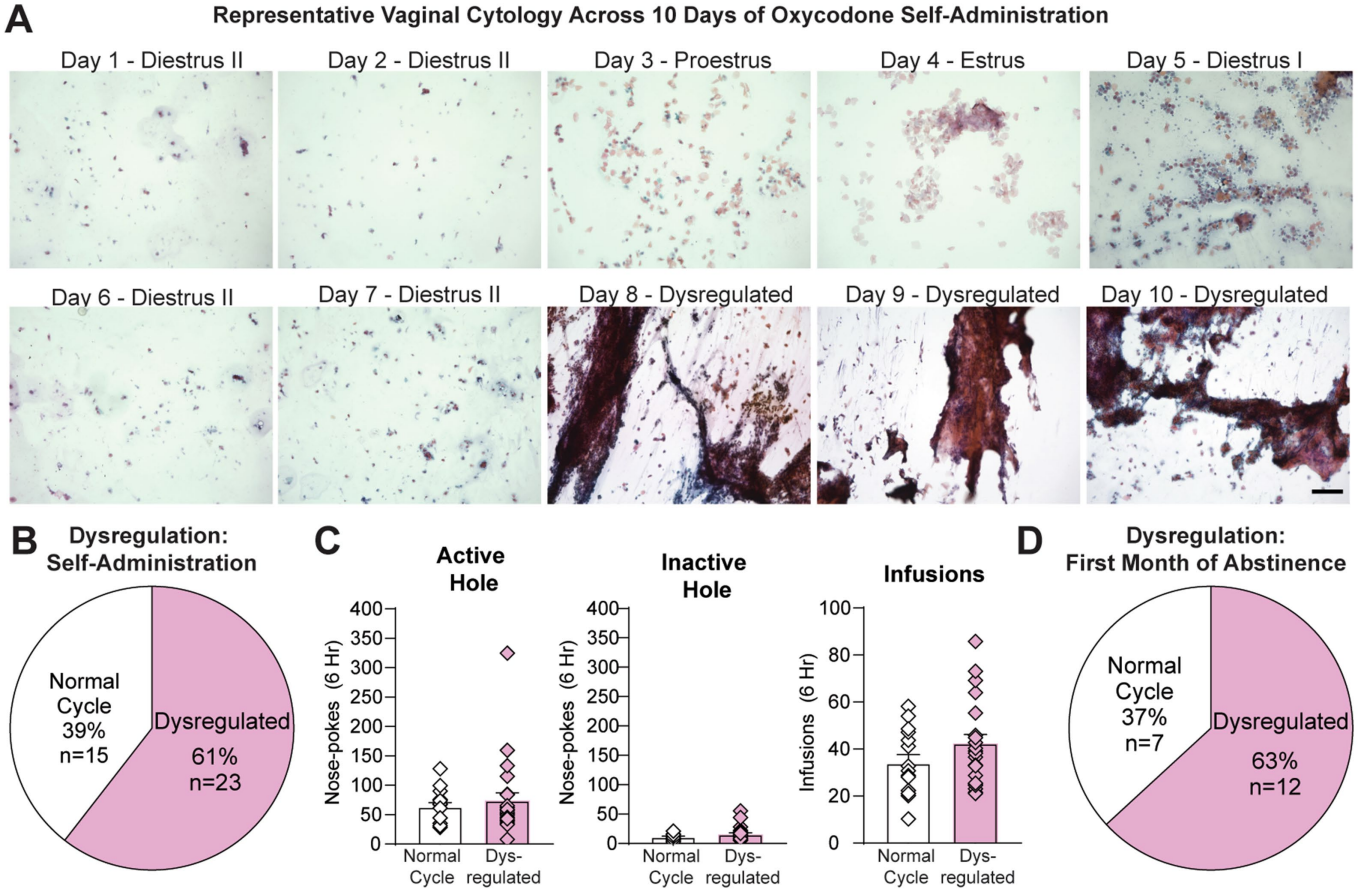
Oxycodone-induced estrous cycle dysregulation. **(A)** Representative images from one female rat across the 10 days of oxycodone self-administration. Following a normal cycle (days 1–4), the animal did not enter estrus for the next 6 days of self-administration (days 5–10), indicative of low hormone levels and anovulation. Note that the proestrus stage shows some cornified cells, as estrus (E) begins the next day. **(B)** During oxycodone self-administration, the majority of animals (*n* = 23 out of 38, 61%) experienced estrous cycle dysregulation, defined as at least one 4–5 day period (the length of an average cycle) in which they did not enter estrus. **(C)** No differences in oxycodone self-administration (active hole and inactive hole responding, infusions obtained) were observed between animals with normal compared to dysregulated cycles. **(D)** During abstinence, the majority of animals remaining (*n* = 12 out of 19, 63%) showed estrous cycle dysregulation. Data are shown as mean ± SEM. Scale bar = 150 μm.

Using vaginal cytology, a well-established technique used to determine estrous cycle stage (e.g., Cora et al., 2015), we assessed the impact of extended-access oxycodone self-administration on the length and pattern of the estrous cycle. Doing so will allow us to more accurately determine the impact of hormonal fluctuations on oxycodone taking and seeking behavior. We began by vaginally swabbing all female rats (*n* = 38) regularly (4 to 5 days) the week prior to self-administration (Figure 1). We observed normal 4 to 5 day cycles in which animals moved through each cycle stage at the approximate length (diestrus, ~2–3 days; proestrus, ~12–14 h; estrus, ~1–2 days; Ajayi and Akhigbe, 2020) (Figure 2A, days 1–4). We then vaginally swabbed them daily during the ten days of self-administration, during which we observed clear estrous cycle dysregulation. Specifically, we found that the majority of animals (23 out of 38 females or 61%) had at least one 4–5 day period (the length of an average cycle) in which they did not enter estrus (Figure 2A, days 5–10 & Figure 2B), indicative of an anovulatory state in which ovarian hormone levels are low (e.g., Huang et al., 1978; Lu et al., 1979; Carlberg and Fregly, 1985). During this period, their dysregulated smears frequently contained significant amounts of mucous and cellular debris along with clumps of cells that often contained a mixture of different cell types. While closest to a diestrus-like state, some abnormal smears would have no clear stage represented (see days 8–10 in Figure 2A). However, patterns of oxycodone self-administration and oxycodone intake were similar between rats that were dysregulated (*n* = 23) and those that were not (*n* = 15) (Figure 2C), indicating that these changes in estrous cyclicity were not due to individual differences in drug intake. Unpaired *t*-tests of average responding across all 10 days revealed no difference between the average number of active (t_36_ = 0.61, *p* = 0.55) and inactive hole nose-pokes (t_36_ = 1.28, *p* = 0.21) and the total number of oxycodone infusions obtained (t_36_ = 1.52, *p* = 0.14) between rats with regular and dysregulated estrous cycles (Figure 2C). While variable from animal to animal and week to week, this dysregulation continued during the first month of abstinence. Animals that remained past days 1–2 (*n* = 19) were vaginally swabbed during abstinence (4–5 consecutive days for several weeks; Figure 1) and we found that 63% of females (12 out of 19) showed at least one consecutive 4 to 5 day period in which they did not enter estrus during the first month of abstinence (Figure 2D). However, after 1 month of abstinence, all rats exhibited normal 4 to 5 day estrous cycles in which they spent ~2 to 3 days in diestrus, ~12–14 h in proestrus and 1–2 days in estrus (Ajayi and Akhigbe, 2020), similar to what was observed prior to oxycodone self-administration or during the first few days of oxycodone self-administration (days 1–4 in Figure 2A). Together these data indicate that, unlike short-access paradigms (Mavrikaki et al., 2017; Vassoler et al., 2018; Hinds et al., 2023), longer-access oxycodone self-administration leads to significant estrous cycle dysregulation in a significant percentage of animals both during self-administration and within the first month of abstinence.

### 3.2 Impact of sex and estrous cycle fluctuations on oxycodone self-administration

**Figure 3:**
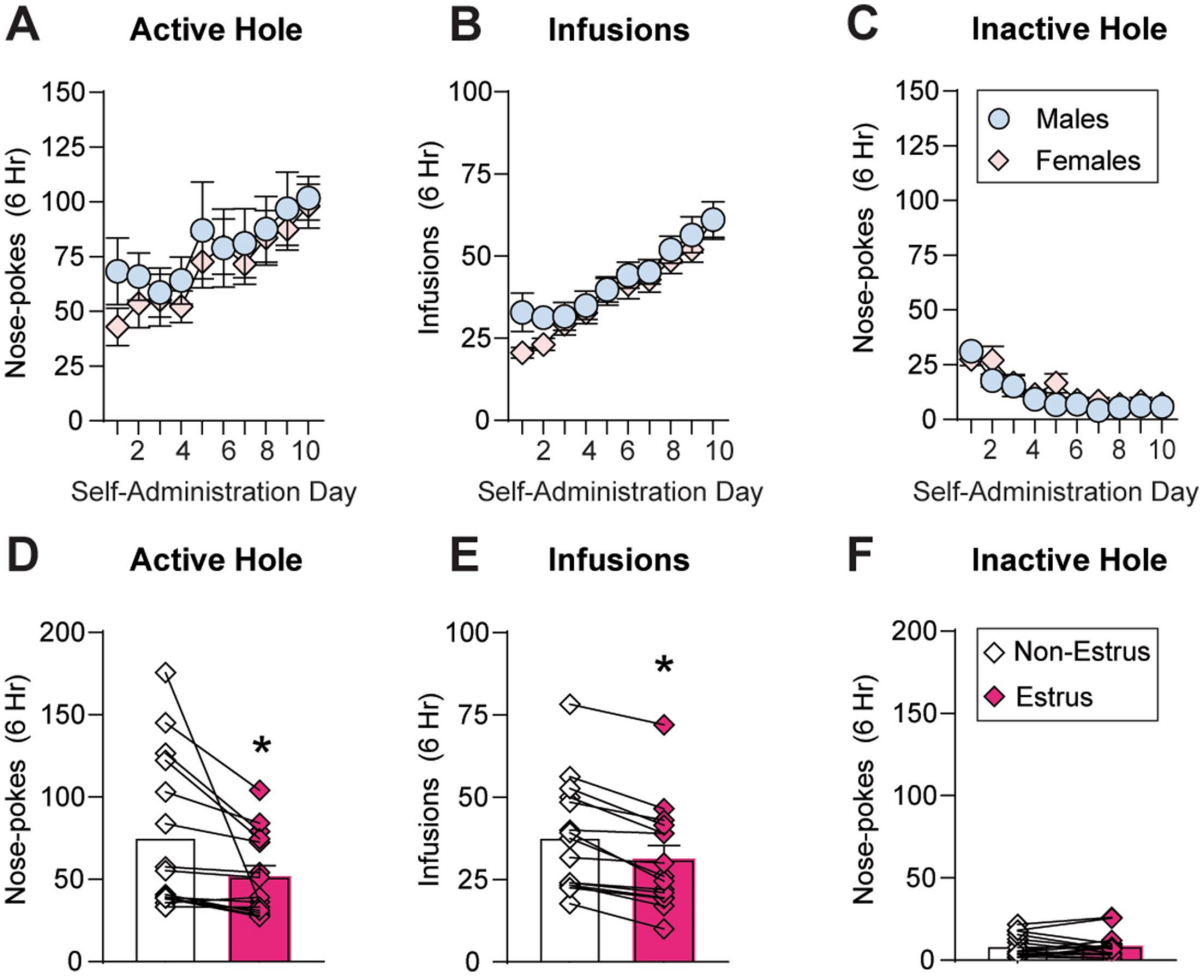
Sex and estrous cycle effects on oxycodone self-administration. **(A–C)** Across the 10 days of self-administration, no group differences between males and females were observed for average active hole responding **(A)**, number of oxycodone infusions obtained **(B)** or inactive hole responding **(C)**. **(D–F)** In animals whose cycles were not dysregulated (*n* = 15), oxycodone self-administration behavior was compared between estrus and non-estrus cycle stages in the same animals across a 5-day period in which intake had stabilized (following acquisition and prior to escalation). During estrus, rats showed a significant reduction in both active hole responding **(D)** and the number of oxycodone infusions obtained **(E)** compared to other cycle stages (non-estrus), but no change in inactive hole responding **(F)**. * *p* < 0.05. Data are shown as mean ± SEM.

As estrous cycle fluctuations were abnormal in the majority of animals (23 out of 38 or 61%) during self-administration (Figure 2B), we first assessed the impact of sex but not estrous cycle on extended-access oxycodone self-administration all of the animals. As expected based on previous reports using a similar oxycodone dose and session length (0.1 mg/kg/infusion, 6 h/d for 10 d; Bossert et al., 2020; Fredriksson et al., 2020), both males (*n* = 19) and females (*n* = 38) showed robust escalation of oxycodone intake over the 10 day period and showed no sex differences in oxycodone self-administration (Figures 3A–C). The between-within ANOVA conducted on these data with sex (male, female) as the between-subjects factor and self-administration day (1–10) as the within-subjects factor revealed no main effect of sex and no interaction between sex and self-administration day on active hole responding (Sex: F_1,55_ = 0.40, *p* = 0.53; Interaction: F_9,495_ = 0.42, *p* = 0.93; Figure 3A), inactive hole responding (Sex: F_1,55_ = 1.48, *p* = 0.23; Interaction: F_9,495_ = 0.895, *p* = 0.53; Figure 3C) or number of infusions obtained (Sex: F_1,55_ = 0.77, *p* = 0.38; Interaction: F_9,495_ = 0.64, *p* = 0.77; Figure 3B). Due to an increase in or escalation of oxycodone intake across the ten-day self-administration period, a significant effect of self-administration day was observed for both active hole responding (F_9,495_ = 7.69, *p* < 0.0001) and the number of oxycodone infusions obtained (F_9,495_ = 25.31, *p* < 0.0001). Due to an expected reduction in inactive hold responding over time, a significant effect of self-administration day on inactive hole responding was also observed (F_9,495_ = 13.63, *p* < 0.0001).

To determine the impact of estrous cycle fluctuations on oxycodone intake, we analyzed self-administration behavior across the cycle in the 15 rats whose cycles were not dysregulated (Figure 2B). Across a 5-day period in which intake had stabilized (following acquisition and prior to escalation, if escalation occurred), active and inactive hole responding as well as oxycodone infusions were compared within the same subjects across different cycle stages. Drug taking was compared in estrus versus other cycle stages (non-estrus), as previous studies have identified changes in drug taking and seeking across different drug classes selectively during estrus, when estradiol and progesterone have just dropped from peak levels and ovulation occurs (e.g., Lynch et al., 2000; Kerstetter et al., 2008; Corbett et al., 2021; Bakhti-Suroosh et al., 2021; Towers et al., 2022). Similar to previous reports of a reduction in oxycodone reinforcement around the time of ovulation (proestrus-estrus) under short-access conditions (Hinds et al., 2023), we observed a significant reduction in both active hole responding (t_14_ = 2.54, *p* = 0.024; Figure 3D) and oxycodone intake (t_14_ = 5.96, *p* < 0.0001; Figure 3E) during estrus compared non-estrus, with no change in inactive hole responding (t_14_ = 0.14, *p* = 0.9889; Figure 3F). Thus, while there were no overall changes in average oxycodone intake between animals whose cycles are regulated or dysregulated (Figure 2C), there were subtle but significant changes in intake across the estrous cycle in regularly cycling females (Figures 3D–F). These findings indicate that, during periods of normal estrous cyclicity and stable intake, a decrease in oxycodone intake is observed during estrus compared to other cycle stages, a finding that is opposite from that observed for psychostimulants like cocaine (e.g., Roberts et al., 1989; Hecht et al., 1999; Lynch et al., 2000).

### 3.3 Sex-dependent changes in incubated cue-induced oxycodone seeking

**Figure 4:**
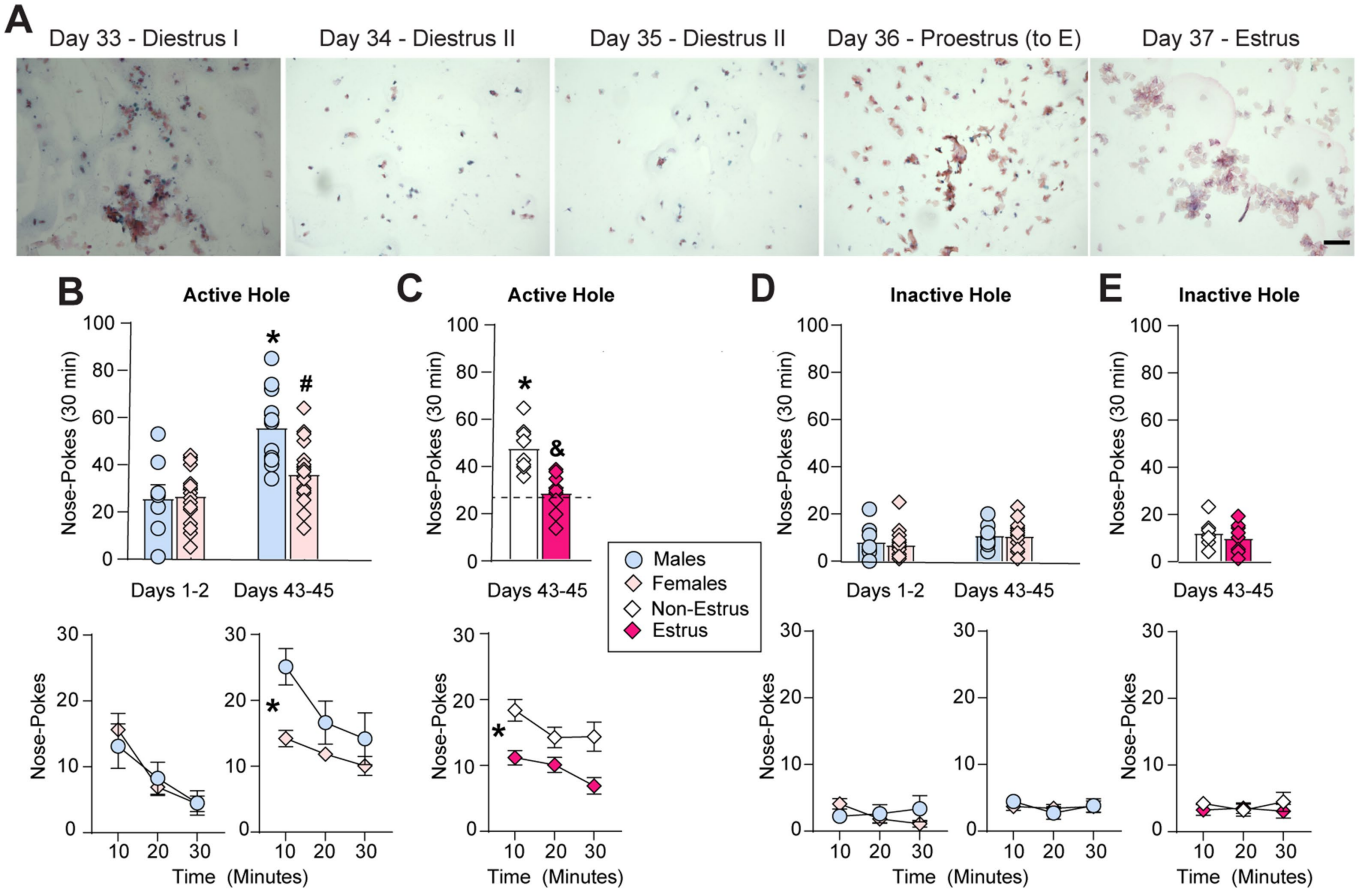
Sex and estrous cycle effects on cue-induced oxycodone seeking behavior. **(A)** Representative images following prolonged abstinence from extended-access oxycodone self-administration from the same rat in Figure 1, showing that normal estrous cyclicity had returned around the time of the second seeking test. Note that the proestrus stage shows some cornified cells, as estrus (E) begins the next day. **(B)** Males showed a significant increase in cue-induced oxycodone seeking in the active hole (previously paired with drug) over days 43–45 compared to days 1–2 (* *p* < 0.05, vs. days 1–2), while females did not. Females also showed a significant reduction in average cue-induced oxycodone seeking compared to males (# *p* < 0.05, top) across the 30 min seeking test (* *p* < 0.05, significant main effect of sex, bottom) over days 43–45. **(C)** This sex differences was driven by a selective reduction in cue-induced oxycodone seeking during estrus but not other cycle stages (non-estrus). Top: * *p* < 0.05, vs. all females on days 1–2 (dashed line); & *p* < 0.05, vs. non-estrus. Bottom: * *p* < 0.05, main effect of estrous cycle. **(D,E)** There was no impact of sex **(D)** or estrous cycle **(E)** on responding in the inactive hole (without consequence) on either test day. Data are shown as mean ± SEM. Scale bar = 150 μm.

Following extended-access oxycodone self-administration, separate groups of animals underwent forced abstinence in their home cages and received cue-induced seeking tests in a drug free state during early (1–2 days) or later (43–45 days) periods of abstinence (Figure 1). While cue-induced oxycodone seeking significantly increased or incubated on days 43–45 (*n* = 11) compared to days 1–2 (*n* = 8) in male rats, seeking or craving did not incubate from days 1–2 (*n* = 19) to 43–45 (*n* = 19) in female rats (Figure 4B). The ANOVA conducted on average active hole (previously paired with drug) nose-pokes with sex (males, females) and test day (1, 44) as the between-subjects factors revealed a significant effect of sex (F_1,53_ = 6.11, *p* = 0.017), a significant effect of test day (F_1,53_ = 26.89, *p* < 0.0001) and a significant interaction between sex and test day (F_1,53_ = 7.51, *p* = 0.008). Tukey post-hoc tests revealed a significant increase in or incubation of cue-induced oxycodone seeking on days 43–45 compared to days 1–2 in males (*, *p* < 0.0001) but not females (*p* = 0.16) and significantly lower cue-induced oxycodone seeking in females compared to males on the later test day (#, *p* = 0.0015; Figure 4B). Similar effects were observed when seeking data were analyzed in 10 min intervals across the 30 min test session. ANOVAs conducted on active hole responding at each time point with sex (males, females) as the between-subjects factor and time (10, 20, and 30 min) as the within-subjects factor revealed no main effect of sex on days 1–2 (F_1,25_ = 0.04, *p* = 0.85) but a significant main effect of sex on days 43–45 (F_1,28_ = 13.9, *p* = 0.0009), when incubation occurred in males but not females (Figure 4B). No interaction between sex and time was observed on either days 1–2 (F_2,50_ = 0.37, *p* = 0.69) or days 43–45 (F_2,56_ = 1.57, *p* = 0.22). A significant effect of time on each seeking test day was observed since, as expected, drug seeking was highest during the first 10 min and gradually decreased across each session (days 1–2: F_2,50_ = 9.91, *p* = 0.0002; days 43–45: F_2,56_ = 6.84, *p* = 0.0022).

No effect of sex was observed on inactive hole (without consequence) responding, indicating that sex selectively influences oxycodone-seeking behavior. The ANOVA conducted on average inactive hole responding revealed no main effect of sex (F_1,53_ = 0.19, *p* = 0.66) or interaction between sex and test day (F_1,53_ = 0.09, *p* = 0.76; Figure 4D). A small but significant effect of test day (F_1,53_ = 4.06, *p* = 0.049) was observed due to a very small increase in inactive hole responding on days 43–45 compared with days 1–2, similar to what we have observed previously in cocaine-exposed rats (Corbett et al., 2021). Similarly, the ANOVA conduced on the time-course of inactive hole responding revealed no significant effect of sex (days 1–2: F_1,25_ = 0.23, *p* = 0.63; days 43–45: F_1,28_ = 0.01, *p* = 0.92) or time (days 1–2: F_2,50_ = 0.68, *p* = 0.51; days 43–45: F_2,56_ = 0.91, *p* = 0.41) and no interaction between sex and time (days 1–2:F_2,50_ = 2.53, *p* = 0.09; days 43–45: F_2,56_ = 0.48, *p* = 0.62; Figure 4D).

### 3.4 Estrous cycle-dependent changes in incubated cue-induced oxycodone seeking

To determine whether the reduction in incubated oxycodone seeking that we observed in female rats compared to male rats on days 43–45 (Figure 4B) was due to changes in hormonal fluctuations across the estrous cycle, we separated the female seeking data (*n* = 19 females) to compare animals in estrus (*n* = 11) and those in other cycle stages (non-estrus, *n* = 8). This comparison has been used previously by both our lab and others as changes in drug seeking behavior are most robust during estrus compared to rats in other cycle stages (non-estrus) for stimulants (Kerstetter et al., 2008; Nicolas et al., 2019; Corbett et al., 2021; Corbett et al., 2023a,b), nicotine (Lynch et al., 2019) and opioids (Bakhti-Suroosh et al., 2021; Towers et al., 2022). Interestingly, a significant reduction in cue-induced oxycodone seeking was observed selectively during the estrus stage of the estrous cycle on days 43–45 in female rats (Figure 4C). The one-way ANOVA conducted on seeking behavior on days 1–2 in all females (as estrous cyclicity was dysregulated at this time-point; Figures 2B,D) and on days 43–45 in females in non-estrus and estrus revealed a significant main effect (F_2,35_ = 12.92, *p* < 0.0001). Tukey post-hoc tests revealed that females in non-estrus incubated on days 43–45 (*, *p* < 0.0001 vs. all females on days 1–2) while females in estrus did not (*p* = 0.94, vs. all females on days 1–2). Importantly, females in estrus showed a significant reduction in cue-induced seeking on days 43–45 compared to females in non-estrus (#, *p* = 0.0006; Figure 4C). Similar effects were observed when seeking data were analyzed in 10 min intervals across the 30 min test session (Figure 4C). The between-within ANOVA conducted on active hole responding on days 43–45 with cycle (non-estrus, estrus) as the between-subjects factor and time (10, 20, and 30 min) as the within-subjects factor revealed a significant main effect of cycle (F_1,17_ = 23.44, *p* = 0.0002) but no interaction between estrous cycle and time (F_2,34_ = 0.88, *p* = 0.43). A significant effect of time was also observed since, as expected, drug seeking was highest during the first 10 min and gradually decreased across each session (F_2,34_ = 4.55, *p* = 0.018). No group differences were observed in average inactive hole responding between females in non-estrus and estrus on days 43–45 (F_2,35_ = 2.473, *p* = 0.099) or across the 30 min seeking test session (cycle: F_1,17_ = 0.64, *p* = 0.44; cycle x time: F_2,34_ = 0.42, *p* = 0.63; time: F_2,34_ = 0.12, *p* = 0.89; Figure 4E). These findings identify a significant reduction in cue-induced oxycodone seeking during estrus following prolonged abstinence, the opposite of what has been observed for nicotine (Lynch et al., 2019), stimulants (Nicolas et al., 2019; Corbett et al., 2021; Corbett et al., 2023a,b) and fentanyl (Bakhti-Suroosh et al., 2021; Towers et al., 2022).

## 4 Discussion

The current study assessed the impact of extended-access oxycodone self-administration on estrous cyclicity as well as the impact of sex and estrous cycle fluctuations, when intact, on oxycodone taking and seeking behavior. Three main findings have emerged from these studies. First, we identified oxycodone-induced estrous cycle dysregulation during both self-administration and the first month of abstinence in the majority (~60%) of the females in this study. This did not appear to be due to differences in oxycodone intake, as self-administration behavior was similar between animals with both regulated and dysregulated cycles. Second, in females whose estrous cycles were not dysregulated (39%), we found a reduction in both active hole responding and the number of oxycodone infusions obtained during estrus compared to other cycle stages (non-estrus). Third, we observed a significant reduction in cue-induced oxycodone seeking following prolonged abstinence in females compared to males that was driven by a reduction in drug seeking during the estrus stage of the cycle. Together these findings (1) highlight the importance of tracking estrous cyclicity during drug self-administration and abstinence and (2) identify a clear impact of hormonal fluctuations across the estrous cycle on oxycodone taking and seeking behavior.

### 4.1 Oxycodone-induced estrous cycle dysregulation

An important finding in the present study is the identification of oxycodone-induced estrous cycle dysregulation observed both during extended-access self-administration and the first month of forced abstinence. This information has allowed us to more accurately assess the impact of estrous cycle fluctuations on oxycodone taking and seeking behavior in the current study (Figures 3, 4). While several studies have identified no impact of short-access oxycodone self-administration (1–2 h) on estrous cycle fluctuations (Mavrikaki et al., 2017; Vassoler et al., 2018; Hinds et al., 2023), less is known regarding how extended-access oxycodone self-administration impacts estrous cyclicity. Previous studies using longer-access paradigms either did not track estrous cycle stages (Bossert et al., 2020; Fredriksson et al., 2020; Kimbrough et al., 2020) or were unable to characterize the impact of oxycodone on estrous cyclicity because they observed cycle dysregulation prior to drug exposure (Olaniran et al., 2023).

Estrous cyclicity is controlled by the hypothalamic–pituitary-gonadal axis, and estradiol and progesterone play a critical role in modulating this feedback loop to ensure proper cycling (reviewed in Becker et al., 2005; Seyfried and Hester, 2012; Scharfman and MacLusky, 2014; Herbison, 2020). In order for ovulation to occur during the estrus stage of the cycle, changes in estradiol and progesterone levels influence the release of gonadotropin-releasing hormone (GnRH) in the hypothalamus (reviewed in Herbison, 2020). This triggers the production of luteinizing hormone (LH) and follicle-stimulating hormone (FSH) in the pituitary gland, which subsequently increases estradiol and progesterone production in the ovaries (Becker et al., 2005; Seyfried and Hester, 2012; Scharfman and MacLusky, 2014; Herbison, 2020). Endogenous and exogenous opioids like oxycodone inhibit this system by binding to opioid receptors in the hypothalamus, thereby decreasing GnRH release and reducing the production of estrogen and progesterone in the ovaries, leading to subsequent cycle irregularities (reviewed in Brennan, 2013). For example, administration of a high dose of morphine (60 mg/kg, i.p.) to intact, cycling female rats blocks ovulation by completely abolishing the release of luteinizing hormone (LH) and reducing the FSH surge (Pang et al., 1977). Consistent with this relationship, we found that the majority of the oxycodone-exposed rats in our study had at least one 4–5 day period in which they did not enter the estrus stage of the cycle, when ovulation normally occurs, indicating low ovarian hormone levels and lack of ovulation or anovulation (Figure 2). Specifically, 23 out of 38 rats (61%) showed this cycle dysregulation over the 10 days of extended-access oxycodone self-administration (Figure 2B). In the animals tested at the later time-point (*n* = 19), this same dysregulation occurred at least once during the first month of abstinence in the majority of the animals (12 out of 19, 63%; Figure 2D).

Importantly, we observed a significant amount of individual variability across subjects in terms of exactly when dysregulation occurred and for how long. We also found that a significant number of animals did not show dysregulated cycles during self-administration (15 out of 38, 39%; Figure 2B) or abstinence (7 out of 19, 37%; Figure 2D), indicating that, similar to our previous reports (Corbett et al., 2021), regular vaginal swabbing alone is not producing cycle dysregulation. We did not observe significant differences in oxycodone intake between animals whose cycles were dysregulated and animals who continued to cycle regularly (Figure 2C), suggesting that the variability was not simply due to the amount of oxycodone the animals self-administered. Similar variability has been observed in humans. For example, while many women who chronically use opioids experience permanent or transient amenorrhea (cycling or menses stops), a smaller number will continue to menstruate (e.g., Santen et al., 1975; Rhodin et al., 2010). Due to this variability, oxycodone-induced estrous cycle dysregulation was only discovered here because we tracked the cycle consecutively (4 to 5 days in a row per week) in animals before, during and after self-administration. In this way, we were able to catch periods of cycle dysregulation that would have been missed if we had only swabbed during or just around periods of behavioral testing, which could have resulted in mischaracterization of cycle stages and inappropriate grouping of animals. Our findings highlight the importance of fully characterizing drug-induced changes in estrous cyclicity across the study to most accurately assess the impact of ovarian hormone fluctuations on oxycodone taking and seeking behavior.

While outside the scope of this study, it is important to note that chronic opioid exposure also leads to hormonal dysregulation in males via similar changes within the hypothalamic pituitary gonadal axis described above. In males, these changes can result in decreased levels of testosterone and infertility (e.g., Daniell, 2008; Seyfried and Hester, 2012; Brennan, 2013). Future studies should examine how extended-access opioid self-administration and forced abstinence impacts gonadal hormone levels in both males and females and how hormonal depletion and replacement of all 3 gonadal hormones (estradiol, progesterone, testosterone) impacts oxycodone taking and seeking behavior in both sexes.

### 4.2 Impact of sex on oxycodone self-administration

Consistent with previous reports using a similar self-administration paradigm (0.1 mg/kg/infusion, fixed ratio 1, 6 h sessions for at least 10 days), we found no sex differences in extended-access oxycodone self-administration on any measures—active and inactive hole responding and number of infusions obtained (Bossert et al., 2020; Fredriksson et al., 2020) (Figures 3A–C). The lack of sex differences in oxycodone self-administration reported here are also consistent with several studies showing no sex differences in shorter-access (1–2 h) oxycodone self-administration (Mavrikaki et al., 2017; Vassoler et al., 2018; Hinds et al., 2023). Studies that have reported sex differences in intravenous oxycodone self-administration differed significantly from the current study in regard to session length, dose, and/or reinforcement schedule. For example, following several days of longer-access (12 h) oxycodone self-administration, one study found that females took more oxycodone than males (Kimbrough et al., 2020). Another study found that, while few sex differences in short-access oxycodone intake emerged across most schedules of reinforcement, a dose–response curve revealed that females did take more oxycodone than males at lower doses (0.01–0.03 mg/kg/infusion) and under a higher schedule of reinforcement than those used here (fixed ratio 5; Mavrikaki et al., 2017). Together, the current findings along with other reports (Mavrikaki et al., 2017; Vassoler et al., 2018; Bossert et al., 2020; Fredriksson et al., 2020; Hinds et al., 2023) generally indicate that under lower reinforcement schedules and/or higher doses such as the conditions used here, males and females show similar levels of intravenous oxycodone intake.

### 4.3 Impact of estrous cycle fluctuations on oxycodone self-administration and cue-induced oxycodone seeking

In a subset of animals whose cycles were not dysregulated during self-administration, we discovered a reduction in extended-access oxycodone self-administration during the estrus stage of the estrous cycle (Figures 3D,E), after estradiol and progesterone levels have peaked and when ovulation occurs. This is generally consistent with recent reports showing that oxycodone reinforcement also decreases during both proestrus and estrus compared to metestrus/diestrus (Hinds et al., 2023). Similarly, heroin intake decreases during proestrus, when estradiol and progesterone are elevated, an effect that is estradiol- but not progesterone-dependent (Ethridge and Smith, 2023). These findings are distinct from the impact of estrous cycle fluctuations on cocaine self-administration, as females in estrus will work harder to self-administer cocaine (Hecht et al., 1999; Lynch et al., 2000) and will consume more cocaine (Lynch et al., 2000), effects that are estradiol-dependent (Roberts et al., 1989; Lynch et al., 2001; Jackson et al., 2006). Therefore, estradiol appears to be impacting the self-administration of opioids and stimulants in opposite ways. A focus for future studies will be to determine what is driving the decrease in opioid intake or responding for opioids observed across the cycle here (Figure 3) and by others (Ethridge and Smith, 2023; Hinds et al., 2023). For example, this decrease could be due to heightened sensitivity to the drug such that less is needed to obtain a reinforcing effect. Conversely, this decrease could be due to a lower reinforcing efficacy of the drug leading to less intake or reduced motivation to obtain the drug.

Similar to intake, we also observed a reduction in cue-induced oxycodone seeking in estrus following prolonged abstinence (days 43–45; Figure 4), an effect which is also opposite of that observed following cocaine exposure and abstinence (Kerstetter et al., 2008; Nicolas et al., 2019; Corbett et al., 2021; Corbett et al., 2023a,b). Importantly, females in estrus on days 43–45 did not show incubation or the time-dependent increase in oxycodone seeking behavior that normally occurs during the first month of forced abstinence and is thought to reflect increased relapse vulnerability (e.g., Grimm et al., 2001; Venniro et al., 2021). These findings differ from recently published findings showing no impact of estrous cycle fluctuations on incubated oxycodone seeking following a significantly shorter abstinence period (2 weeks; Olaniran et al., 2023). However, Olaniran and colleagues observed estrous cycle dysregulation prior to the start of oxycodone self-administration. They also found a significant impact of oxycodone exposure on estrous cyclicity (time spent in each cycle stage), both of which may have masked any estrous cycle-dependent changes in oxycodone seeking. Alternatively, the impact of estrous cycle fluctuations on cue-induced oxycodone seeking could be time-dependent and may not emerge within the first 2 weeks of abstinence. Indeed, we have found that estrous cycle-dependent changes in incubated cocaine seeking intensifies between 2 and 7 weeks of abstinence (Corbett et al., 2021), albeit in the opposite direction from the effects observed here following oxycodone exposure. Unfortunately, the estrous cycle dysregulation here observed during the first month of abstinence (Figure 2) prevented us from accurately assessing the time course of estrous cycle-dependent changes in incubated oxycodone seeking.

Other studies investigating the impact of estrous cycle fluctuations on opioid seeking using other opioids and/or other relapse models have yielded mixed results. One report found that the administration of exogenous estradiol led to more robust extinction and a consequential reduction in heroin seeking in freely cycling females (Vazquez et al., 2020). While this generally fits with our findings, our seeking test data are not suggestive of changes in extinction learning, as we did not observe an interaction between estrous cycle stage and time (Figure 4B). However, others found an increase in cue-induced reinstatement of previously extinguished fentanyl seeking behavior in estrus compared to non-estrus females (Bakhti-Suroosh et al., 2021; Towers et al., 2022). An increase in oxycodone conditioned place preference was also observed during estrus compared to other cycle stages (Babb et al., 2023). Additional studies also found no impact of estrous cycle fluctuations on cue-induced reinstatement of oxycodone seeking (Hinds et al., 2023) or incubated heroin craving (Mayberry et al., 2022). While session length, dose, drug, behavioral paradigm and estrous cyclicity may all contribute to these disparate findings, future studies are needed to fully characterize and interpret the impact of ovarian hormones on opioid seeking reward-related behavior across different paradigms.

Another important future direction is to identify the cellular mechanisms underlying the distinct effects of estrous cycle fluctuations on taking and seeking behavior in stimulant- and opioid-exposed animals. While estradiol is known to impact behavioral responding to cocaine by altering cocaine-induced dopamine release in the nucleus accumbens (NAc; Becker, 2016) and via changes in glutamate receptor-dependent signaling in the NAc (Tonn Eisinger et al., 2018), the mechanisms underlying estradiol-dependent changes in opioid reinforcement have not yet been explored. However, it is known that elevated estradiol levels increase endogenous opioid tone (e.g., peptide and opioid receptor levels) in the hypothalamus to initiate changes in sexual receptivity during proestrus and estrus (reviewed in Micevych and Meisel, 2017). While this relationship has not been explored as extensively in the NAc, increases in endogenous opioid peptides have been observed in the NAc in proestrus/estrus compared to metestrus/diestrus (Roman et al., 2006). Ovariectomized females also showed a reduction in preproenkephalin mRNA levels in the NAc and striatum which were restored following 2 weeks estradiol treatment (Le Saux and Di Paolo, 2005). An increase in opioid receptor mRNA and protein expression in the striatum has also been observed following co-administration of opioids and ovarian hormones (Teodorov et al., 2014; Cruz et al., 2015). One intriguing possibility is that estrous cycle-dependent increases in endogenous opioid tone are occurring within the NAc to influence the changes in oxycodone taking and seeking that we (Figures 3, 4) and others (Hinds et al., 2023) have observed across the cycle. Interestingly, a role for endogenous NAc opioid signaling in mediating incubated cue-induced cocaine craving has also been identified. Dikshtein and colleagues have shown that increased *β*-endorphin levels in the NAc are associated with reduced incubated cue-induced cocaine seeking in male rats (Dikshtein et al., 2013), indicating that this could be a shared mechanism influencing craving across both substances but one that could theoretically be impacted differently by ovarian hormones across the two drug classes. To fully investigate these mechanisms, future studies should be conducted in intact, freely cycling rats both in the absence and presence of hormone receptor antagonists as well as following hormonal depletion and replacement. Future studies should also address how the timing of hormonal fluctuations across the estrous cycle (e.g., comparing the impact of hormone receptor antagonism in proestrus vs. estrus) contributes to the estrous cycle-dependent changes in oxycodone taking and seeking behavior observed here. Together this knowledge may inform and improve prescribing practices for drugs like oxycodone by taking an individual’s hormonal status into account, which may in turn reduce their potential abuse liability.

## Data Availability

The raw data supporting the conclusions of this article will be made available by the authors, without undue reservation.

## References

[ref1] Ajayi A. F. Akhigbe R. E. (2020). Staging of the estrous cycle and induction of estrus in experimental rodents: an update. Fertil. Res. Pract. 6:5. doi: 10.1186/s40738-020-00074-3, PMID: 32190339 PMC7071652

[ref2] Babb J. A. Constantino N. J. Kaplan G. B. Chartoff E. H. (2023). Estrous cycle dependent expression of oxycodone conditioned reward in rats. Sci. Rep. 13:13946. doi: 10.1038/s41598-023-40971-3, PMID: 37626154 PMC10457365

[ref3] Bakhti-Suroosh A. Towers E. B. Lynch W. J. (2021). A buprenorphine-validated rat model of opioid use disorder optimized to study sex differences in vulnerability to relapse. Psychopharmacology 238, 1029–1046. doi: 10.1007/s00213-020-05750-2, PMID: 33404740 PMC7786148

[ref4] Becker J. B. (2016). Sex differences in addiction. Dialogues Clin. Neurosci. 18, 395–402. doi: 10.31887/DCNS.2016.18.4/jbecker, PMID: 28179811 PMC5286725

[ref5] Becker J. B. Arnold A. P. Berkley K. J. Blaustein J. D. Eckel L. A. Hampson E.. (2005). Strategies and methods for research on sex differences in brain and behavior. Endocrinology 146, 1650–1673. doi: 10.1210/en.2004-1142, PMID: 15618360

[ref6] Bossert J. M. Kiyatkin E. A. Korah H. Hoots J. K. Afzal A. Perekopskiy D.. (2020). In a rat model of opioid maintenance, the G protein-biased mu opioid receptor agonist TRV130 decreases relapse to oxycodone seeking and taking and prevents oxycodone-induced brain hypoxia. Biol. Psychiatry 88, 935–944. doi: 10.1016/j.biopsych.2020.02.014, PMID: 32305216 PMC7483192

[ref7] Brennan M. J. (2013). The effect of opioid therapy on endocrine function. Am. J. Med. 126, S12–S18. doi: 10.1016/j.amjmed.2012.12.001 23414717

[ref8] Carlberg K. A. Fregly M. J. (1985). Disruption of estrous cycles in exercise-trained rats. Proc. Soc. Exp. Biol. Med. 179, 21–24. doi: 10.3181/00379727-179-42058, PMID: 4039441

[ref9] Carroll M. E. Lynch W. J. (2016). How to study sex differences in addiction using animal models. Addict. Biol. 21, 1007–1029. doi: 10.1111/adb.12400, PMID: 27345022 PMC4970981

[ref10] Carroll M. E. Lynch W. J. Roth M. E. Morgan A. D. Cosgrove K. P. (2004). Sex and estrogen influence drug abuse. Trends Pharmacol. Sci. 25, 273–279. doi: 10.1016/j.tips.2004.03.011 15120494

[ref11] Cora M. C. Kooistra L. Travlos G. (2015). Vaginal cytology of the laboratory rat and mouse: review and criteria for the staging of the estrous cycle using stained vaginal smears. Toxicol. Pathol. 43, 776–793. doi: 10.1177/0192623315570339 25739587 PMC11504324

[ref12] Corbett C. M. Dunn E. Loweth J. A. (2021). Effects of sex and estrous cycle on the time course of incubation of Cue-induced craving following extended-access cocaine self-administration. eNeuro 8, ENEURO.0054–ENEU21.2021. doi: 10.1523/ENEURO.0054-21.2021, PMID: 34290059 PMC8362687

[ref13] Corbett C. M. Miller E. N. D. Loweth J. A. (2023a). mGlu5 inhibition in the basolateral amygdala prevents estrous cycle-dependent changes in cue-induced cocaine seeking. Addict. Neurosci. 5:100055. doi: 10.1016/j.addicn.2022.100055, PMID: 36778664 PMC9915145

[ref14] Corbett C. M. Miller E. N. D. Wannen E. E. Rood B. D. Chandler D. J. Loweth J. A. (2023b). Cocaine exposure increases excitatory synaptic transmission and intrinsic excitability in the basolateral amygdala in male and female rats and across the estrous cycle. Neuroendocrinology 113, 1127–1139. doi: 10.1159/000531351, PMID: 37271140 PMC10623393

[ref15] Cruz W. S. Pereira L. A. Cezar L. C. Camarini R. Felicio L. F. Bernardi M. M.. (2015). Role of steroid hormones and morphine treatment in the modulation of opioid receptor gene expression in brain structures in the female rat. Springerplus 4:355. doi: 10.1186/s40064-015-1021-8, PMID: 26191482 PMC4503706

[ref16] Daniell H. W. (2008). Opioid endocrinopathy in women consuming prescribed sustained-action opioids for control of nonmalignant pain. J. Pain 9, 28–36. doi: 10.1016/j.jpain.2007.08.005, PMID: 17936076

[ref17] Dickson-Gomez J. Krechel S. Spector A. Weeks M. Ohlrich J. Green Montaque H. D.. (2022). The effects of opioid policy changes on transitions from prescription opioids to heroin, fentanyl and injection drug use: a qualitative analysis. Subst. Abuse Treat. Prev. Policy 17:55. doi: 10.1186/s13011-022-00480-4, PMID: 35864522 PMC9306091

[ref18] Dikshtein Y. Barnea R. Kronfeld N. Lax E. Roth-Deri I. Friedman A.. (2013). beta-endorphin via the delta opioid receptor is a major factor in the incubation of cocaine craving. Neuropsychopharmacology 38, 2508–2514. doi: 10.1038/npp.2013.155, PMID: 23800967 PMC3799071

[ref19] Ethridge S. B. Smith M. A. (2023). Estradiol and mu opioid-mediated reward: the role of estrogen receptors in opioid use. Addict. Neurosci. 9:100139. doi: 10.1016/j.addicn.2023.100139, PMID: 38155959 PMC10753849

[ref20] Fredriksson I. Applebey S. V. Minier-Toribio A. Shekara A. Bossert J. M. Shaham Y. (2020). Effect of the dopamine stabilizer (−)-OSU6162 on potentiated incubation of opioid craving after electric barrier-induced voluntary abstinence. Neuropsychopharmacology 45, 770–779. doi: 10.1038/s41386-020-0602-6, PMID: 31905372 PMC7075949

[ref21] Fulenwider H. D. Nennig S. E. Hafeez H. Price M. E. Baruffaldi F. Pravetoni M.. (2020). Sex differences in oral oxycodone self-administration and stress-primed reinstatement in rats. Addict. Biol. 25:e12822. doi: 10.1111/adb.12822, PMID: 31830773 PMC7289656

[ref22] Glynn R. M. Rosenkranz J. A. Wolf M. E. Caccamise A. Shroff F. Smith A. B.. (2018). Repeated restraint stress exposure during early withdrawal accelerates incubation of cue-induced cocaine craving. Addict. Biol. 23, 80–89. doi: 10.1111/adb.12475, PMID: 27859963 PMC5426993

[ref23] Goetz T. G. Becker J. B. Mazure C. M. (2021). Women, opioid use and addiction. FASEB J. 35:e21303. doi: 10.1096/fj.202002125R, PMID: 33433026

[ref24] Grimm J. W. Hope B. T. Wise R. A. Shaham Y. (2001). Neuroadaptation. Incubation of cocaine craving after withdrawal. Nature 412, 141–142. doi: 10.1038/35084134, PMID: 11449260 PMC2889613

[ref25] Hecht G. S. Spear N. E. Spear L. P. (1999). Changes in progressive ratio responding for intravenous cocaine throughout the reproductive process in female rats. Dev. Psychobiol. 35, 136–145. doi: 10.1002/(SICI)1098-2302(199909)35:2<136::AID-DEV6>3.0.CO;2-K, PMID: 10461127

[ref26] Herbison A. E. (2020). A simple model of estrous cycle negative and positive feedback regulation of GnRH secretion. Front. Neuroendocrinol. 57:100837. doi: 10.1016/j.yfrne.2020.100837, PMID: 32240664

[ref27] Hinds N. M. Wojtas I. D. Gallagher C. A. Corbett C. M. Manvich D. F. (2023). Effects of sex and estrous cycle on intravenous oxycodone self-administration and the reinstatement of oxycodone-seeking behavior in rats. Front. Behav. Neurosci. 17:1143373. doi: 10.1101/2023.06.02.543393, PMID: 37465001 PMC10350507

[ref28] Huang H. H. Steger R. W. Bruni J. F. Meites J. (1978). Patterns of sex steroid and gonadotropin secretion in aging female rats. Endocrinology 103, 1855–1859. doi: 10.1210/endo-103-5-1855 570913

[ref29] Jackson L. R. Robinson T. E. Becker J. B. (2006). Sex differences and hormonal influences on acquisition of cocaine self-administration in rats. Neuropsychopharmacology 31, 129–138. doi: 10.1038/sj.npp.1300778 15920500

[ref30] Kerstetter K. A. Aguilar V. R. Parrish A. B. Kippin T. E. (2008). Protracted time-dependent increases in cocaine-seeking behavior during cocaine withdrawal in female relative to male rats. Psychopharmacology 198, 63–75. doi: 10.1007/s00213-008-1089-8, PMID: 18265959

[ref31] Kimbrough A. Kononoff J. Simpson S. Kallupi M. Sedighim S. Palomino K.. (2020). Oxycodone self-administration and withdrawal behaviors in male and female Wistar rats. Psychopharmacology 237, 1545–1555. doi: 10.1007/s00213-020-05479-y, PMID: 32114633 PMC7269712

[ref32] Knouse M. C. Briand L. A. (2021). Behavioral sex differences in cocaine and opioid use disorders: the role of gonadal hormones. Neurosci. Biobehav. Rev. 128, 358–366. doi: 10.1016/j.neubiorev.2021.06.038, PMID: 34214512 PMC8363946

[ref33] Le Saux M. Di Paolo T. (2005). Chronic estrogenic drug treatment increases preproenkephalin mRNA levels in the rat striatum and nucleus accumbens. Psychoneuroendocrinology 30, 251–260. doi: 10.1016/j.psyneuen.2004.08.002, PMID: 15511599

[ref34] Lu L. Grimm J. W. Hope B. T. Shaham Y. (2004). Incubation of cocaine craving after withdrawal: a review of preclinical data. Neuropharmacology 47, 214–226. doi: 10.1016/j.neuropharm.2004.06.027, PMID: 15464139

[ref35] Lu K. H. Hopper B. R. Vargo T. M. Yen S. S. (1979). Chronological changes in sex steroid, gonadotropin and prolactin secretions in aging female rats displaying different reproductive states. Biol. Reprod. 21, 193–203. doi: 10.1095/biolreprod21.1.193, PMID: 573635

[ref36] Lynch W. J. Arizzi M. N. Carroll M. E. (2000). Effects of sex and the estrous cycle on regulation of intravenously self-administered cocaine in rats. Psychopharmacology 152, 132–139. doi: 10.1007/s002130000488, PMID: 11057516

[ref37] Lynch W. J. Roth M. E. Mickelberg J. L. Carroll M. E. (2001). Role of estrogen in the acquisition of intravenously self-administered cocaine in female rats. Pharmacol. Biochem. Behav. 68, 641–646. doi: 10.1016/s0091-3057(01)00455-5, PMID: 11526960

[ref38] Lynch W. J. Tan L. Narmeen S. Beiter R. Brunzell D. H. (2019). Exercise or saccharin during abstinence block estrus-induced increases in nicotine-seeking. Physiol. Behav. 203, 33–41. doi: 10.1016/j.physbeh.2017.10.026, PMID: 29080668 PMC5927845

[ref39] Mack K. A. J. C. M. Paulozzi L. J. Centers for Disease, ControlPrevention (2013). Vital signs: overdoses of prescription opioid pain relievers and other drugs among women--United States, 1999–2010. MMWR Morb. Mortal Wkly. Rep. 62, 537–542. Available at: https://www.ncbi.nlm.nih.gov/pubmed/23820967 23820967 PMC4604783

[ref40] Maher E. E. Strzelecki A. M. Weafer J. J. Gipson C. D. (2023). The importance of translationally evaluating steroid hormone contributions to substance use. Front. Neuroendocrinol. 69:101059. doi: 10.1016/j.yfrne.2023.101059, PMID: 36758769 PMC10182261

[ref41] Mavrikaki M. Pravetoni M. Page S. Potter D. Chartoff E. (2017). Oxycodone self-administration in male and female rats. Psychopharmacology 234, 977–987. doi: 10.1007/s00213-017-4536-6, PMID: 28127624 PMC7250466

[ref42] Mayberry H. L. DeSalvo H. A. Bavley C. C. Downey S. H. Lam C. Kunta C.. (2022). Opioid and sucrose craving are accompanied by unique behavioral and affective profiles after extended abstinence in male and female rats. eNeuro 9, ENEURO.0515–ENEU21.2022. doi: 10.1523/ENEURO.0515-21.2022, PMID: 35241453 PMC9007407

[ref43] Micevych P. E. Meisel R. L. (2017). Integrating neural circuits controlling female sexual behavior. Front. Syst. Neurosci. 11:42. doi: 10.3389/fnsys.2017.00042, PMID: 28642689 PMC5462959

[ref44] Moran-Santa Maria M. M. Flanagan J. Brady K. (2014). Ovarian hormones and drug abuse. Curr. Psychiatry Rep. 16:511. doi: 10.1007/s11920-014-0511-7, PMID: 25224609 PMC4439205

[ref45] Munshi S. Rosenkranz J. A. Caccamise A. Wolf M. E. Corbett C. M. Loweth J. A. (2021). Cocaine and chronic stress exposure produce an additive increase in neuronal activity in the basolateral amygdala. Addict. Biol. 26:e12848. doi: 10.1111/adb.12848, PMID: 31750602 PMC7510484

[ref46] Nicolas C. Russell T. I. Pierce A. F. Maldera S. Holley A. You Z. B.. (2019). Incubation of cocaine craving after intermittent-access self-administration: sex differences and estrous cycle. Biol. Psychiatry 85, 915–924. doi: 10.1016/j.biopsych.2019.01.015 30846301 PMC6534474

[ref47] Olaniran A. Altshuler R. D. Burke M. A. M. Lin H. Firlie J. Linshitz I.. (2023). Role of oestrous cycle and orbitofrontal cortex in oxycodone seeking after 15-day abstinence in female rats. Addict. Biol. 28:e13325. doi: 10.1111/adb.13325, PMID: 37753563

[ref48] Pang C. N. Zimmermann E. Sawyer C. H. (1977). Morphine inhibition of the preovulatory surges of plasma luteinizing hormone and follicle stimulating hormone in the rat. Endocrinology 101, 1726–1732. doi: 10.1210/endo-101-6-1726, PMID: 338290

[ref49] Peart D. R. Andrade A. K. Logan C. N. Knackstedt L. A. Murray J. E. (2022). Regulation of cocaine-related behaviours by estrogen and progesterone. Neurosci. Biobehav. Rev. 135:104584. doi: 10.1016/j.neubiorev.2022.104584, PMID: 35189163

[ref50] Rhodin A. Stridsberg M. Gordh T. (2010). Opioid endocrinopathy: a clinical problem in patients with chronic pain and long-term oral opioid treatment. Clin. J. Pain 26, 374–380. doi: 10.1097/AJP.0b013e3181d1059d, PMID: 20473043

[ref51] Roberts D. C. Loh E. A. Vickers G. (1989). Self-administration of cocaine on a progressive ratio schedule in rats: dose-response relationship and effect of haloperidol pretreatment. Psychopharmacology 97, 535–538. doi: 10.1007/BF00439560, PMID: 2498950

[ref52] Roman E. Ploj K. Gustafsson L. Meyerson B. J. Nylander I. (2006). Variations in opioid peptide levels during the estrous cycle in Sprague-Dawley rats. Neuropeptides 40, 195–206. doi: 10.1016/j.npep.2006.01.004, PMID: 16540166

[ref53] Santen F. J. Sofsky J. Bilic N. Lippert R. (1975). Mechanism of action of narcotics in the production of menstrual dysfunction in women. Fertil. Steril. 26, 538–548. doi: 10.1016/S0015-0282(16)41173-8, PMID: 236938

[ref54] Scharfman H. E. MacLusky N. J. (2014). Sex differences in the neurobiology of epilepsy: a preclinical perspective. Neurobiol. Dis. 72 Pt B, 180–192. doi: 10.1016/j.nbd.2014.07.004, PMID: 25058745 PMC4252793

[ref55] Seyfried O. Hester J. (2012). Opioids and endocrine dysfunction. Br. J. Pain 6, 17–24. doi: 10.1177/2049463712438299, PMID: 26516462 PMC4590093

[ref56] Silver E. R. Hur C. (2020). Gender differences in prescription opioid use and misuse: implications for men's health and the opioid epidemic. Prev. Med. 131:105946. doi: 10.1016/j.ypmed.2019.105946, PMID: 31816359

[ref57] Smyth B. P. Barry J. Keenan E. Ducray K. (2010). Lapse and relapse following inpatient treatment of opiate dependence. Ir. Med. J. 103, 176–179. Available at: https://www.ncbi.nlm.nih.gov/pubmed/20669601 20669601

[ref58] Teodorov E. Camarini R. Bernardi M. M. Felicio L. F. (2014). Treatment with steroid hormones and morphine alters general activity, sexual behavior, and opioid gene expression in female rats. Life Sci. 104, 47–54. doi: 10.1016/j.lfs.2014.03.021 24699004

[ref59] Tonn Eisinger K. R. Gross K. S. Head B. P. Mermelstein P. G. (2018). Interactions between estrogen receptors and metabotropic glutamate receptors and their impact on drug addiction in females. Horm. Behav. 104, 130–137. doi: 10.1016/j.yhbeh.2018.03.001, PMID: 29505763 PMC6131090

[ref60] Towers E. B. Setaro B. Lynch W. J. (2022). Sex- and dose-dependent differences in the development of an addiction-like phenotype following extended-access fentanyl self-administration. Front. Pharmacol. 13:841873. doi: 10.3389/fphar.2022.841873 35370634 PMC8968863

[ref61] Vassoler F. M. Oranges M. L. Toorie A. M. Byrnes E. M. (2018). Oxycodone self-administration during pregnancy disrupts the maternal-infant dyad and decreases midbrain OPRM1 expression during early postnatal development in rats. Pharmacol. Biochem. Behav. 173, 74–83. doi: 10.1016/j.pbb.2018.07.009, PMID: 30055180 PMC6126918

[ref62] Vazquez M. Frazier J. H. Reichel C. M. Peters J. (2020). Acute ovarian hormone treatment in freely cycling female rats regulates distinct aspects of heroin seeking. Learn. Mem. 27, 6–11. doi: 10.1101/lm.050187.119, PMID: 31843977 PMC6919190

[ref63] Venniro M. Reverte I. Ramsey L. A. Papastrat K. M. D'Ottavio G. Milella M. S.. (2021). Factors modulating the incubation of drug and non-drug craving and their clinical implications. Neurosci. Biobehav. Rev. 131, 847–864. doi: 10.1016/j.neubiorev.2021.09.050, PMID: 34597716 PMC8931548

[ref64] Vuong C. Van Uum S. H. O'Dell L. E. Lutfy K. Friedman T. C. (2010). The effects of opioids and opioid analogs on animal and human endocrine systems. Endocr. Rev. 31, 98–132. doi: 10.1210/er.2009-0009, PMID: 19903933 PMC2852206

[ref65] Weiss R. D. Potter J. S. Fiellin D. A. Byrne M. Connery H. S. Dickinson W.. (2011). Adjunctive counseling during brief and extended buprenorphine-naloxone treatment for prescription opioid dependence: a 2-phase randomized controlled trial. Arch. Gen. Psychiatry 68, 1238–1246. doi: 10.1001/archgenpsychiatry.2011.121, PMID: 22065255 PMC3470422

[ref66] Wolf M. E. (2016). Synaptic mechanisms underlying persistent cocaine craving. Nat. Rev. Neurosci. 17, 351–365. doi: 10.1038/nrn.2016.39, PMID: 27150400 PMC5466704

[ref67] Woody G. E. Poole S. A. Subramaniam G. Dugosh K. Bogenschutz M. Abbott P.. (2008). Extended vs short-term buprenorphine-naloxone for treatment of opioid-addicted youth: a randomized trial. JAMA 300, 2003–2011. doi: 10.1001/jama.2008.574, PMID: 18984887 PMC2610690

